# A Systematic Review and Meta-Analysis of Caudal Block as Compared to Noncaudal Regional Techniques for Inguinal Surgeries in Children

**DOI:** 10.1155/2014/890626

**Published:** 2014-08-05

**Authors:** Harsha Shanthanna, Balpreet Singh, Gordon Guyatt

**Affiliations:** ^1^Department of Anesthesiology, McMaster University, St. Joseph's Healthcare Hamilton, 50 Charlton Avenue East, Hamilton, ON, Canada L8N 4A6; ^2^IWK Health Centre and Dalhousie University, Halifax, NS, Canada B3K 6R8; ^3^Department of Clinical Epidemiology and Biostatistics, McMaster University, Hamilton, ON, Canada L8N 4A6

## Abstract

This systematic review and meta-analysis were designed to compare the analgesic effectiveness and adverse effects with the use of caudal analgesia as compared to noncaudal regional analgesia techniques in children undergoing inguinal surgeries. MEDLINE, EMBASE, and CENTRAL (Cochrane) databases were searched for randomized control trials published in English language from 1946 up to 2013. Use of rescue analgesia and adverse effects were considered as primary and secondary outcomes, respectively. Outcomes were pooled using random effects model and reported as risk ratio (RR) with 95% CI. Out of 3240 hits and 24 reports for final selection, 17 were included in this review. Caudal analgesia was found to be better in both early (RR = 0.81 [0.66, 0.99], *P* = 0.04) and late (RR = 0.81 [0.69, 0.96], *P* = 0.01) periods, but with a significant risk of motor block and urinary retention. According to GRADE, the quality of evidence was moderate. Although potentially superior, caudal analgesia increases the chance of motor block and urinary retention. There are limited studies to demonstrate that the technical superiority using ultrasound translates into better clinical success with the inguinal nerve blocks.

## 1. Introduction

The most commonly performed inguinal surgeries in children include inguinal hernia repair with or without orchidopexy (orchiopexy) [1]. For postoperative pain with these surgeries, a regional analgesic modality such as caudal analgesia (CA), inguinal and iliohypogastric nerve block (INB), or local infiltration (INF) is combined with a general anaesthetic (GA). When compared to intravenous (IV) opioids, regional techniques reduce the risk of side effects such as somnolence, respiratory depression, emesis, and ileus [2]. Caudal block (CB) involves the introduction of local anaesthetic (LA) into the caudal epidural space. It requires the child to be positioned appropriately and is a common practice to administer under deep sedation or a GA. It can cause complications such as needle trauma, infection, haematoma, and inadvertent subarachnoid or intravascular injection of the LA [3]. Other associated adverse effects can include urinary retention and possible motor blockade. INB-including inguinal and iliohypogastric nerve blocks can provide effective ipsilateral analgesia. A single injection often blocks both the nerves, as they lie quite close to each other, side by side. Landmark techniques can result in technical failure in up to 20% of children. This can be improved by using ultrasound guidance [4]. They possibly do not affect the pain caused by visceral manipulation. INF of the wound can be done by the anaesthesiologist or the surgeon. This potentially effective, but minimally invasive procedure could offer the advantage of lower costs, time, and risks [5]. Other interventions which have been compared to caudal analgesia include paravertebral block (PVB) and TAP (transverse abdominal plane) block. Although CB may be considered as the most potent technique, it requires trained personnel and added cost; potentially it could expose children to higher risks without any superiority in clinical analgesia. Ultrasound (US) has been shown to improve the technical efficiency and success rate of regional blocks [4]. It is not established whether this translates into comparative clinical success. In clinical practice, there is still no conclusive evidence to prefer one technique over the other in consideration of their efficacy and side effects. The main objective of this review is to perform a systematic review and meta-analysis of the existing evidence to compare the analgesic efficacy and side effects with the use of caudal analgesia as compared to other noncaudal regional analgesia techniques in children undergoing inguinal surgeries.

## 2. Methodology

We performed a comprehensive search in MEDLINE, EMBASE, and Cochrane databases for randomized controlled trials (RCT) in English language (Appendix A). This was complemented by other databases, relevant conference proceedings, and hand check of reference lists of reviews and included RCT. Our selection criteria using the PICOT format are as follows: participants: children (0–12 years) undergoing inguinal surgeries; intervention: CB (without adjuvants); comparators: noncaudal regional techniques; outcome: effectiveness of analgesia assessed using a categorical outcome reporting as the number of children needing rescue analgesia; time point: early (<4 hrs) and late (4–24 hrs). Trials with various comparator techniques such as INB, INF, or a combination of these interventions were included. Trials using adjuvants other than epinephrine (such as ketamine, clonidine) or CB in combination with another technique were excluded. Studies with outcome reporting using “only pain scales” (continuous) were separately reported without combining them in the possible pooled estimate. As secondary outcomes, clinically relevant adverse effects as related to treatments were considered: motor block (MB), urinary retention (UR), nausea-vomiting (NV), infection at the injection site, and delayed discharge (DD). The study selection was done independently by the first two authors, and a final agreement score was calculated using a quadratic kappa weighting. Risk of bias was assessed using the Cochrane risk of bias tool. Considering the nature of interventions, blinding of physicians may not be possible. Hence, only participant blinding was considered necessary to be identified as having a low risk of bias. We considered a loss to follow-up (LTFU) of 10% or more as the threshold for attrition bias. Funnel plot was used to look for any publication bias. Considering the clinically heterogeneous comparator techniques, we decided a priori to subgroup them into CB versus INB, CB versus INF, CB versus combined INB and INF, and CB versus others. Other potential sources of heterogeneity considered were concentration of LA, timing of interventions as related to surgery, and the use of image guidance.

### 2.1. Summary Measures and Synthesis of Results

For the primary outcome, the proportions of children needing rescue analgesia were compared. Rescue analgesia was considered as the administration of an analgesic medication to control pain with or without agitation. Outcomes at the 2 time points, early (<4 hrs) and late (4 hrs–24 hrs), were analysed separately as a pooled estimate for all the studies and also individually within each group. For secondary outcomes, adverse effects in each category were compared as proportion of patients. For both, outcomes were pooled and reported as relative risks with 95% CI.

Synthesis was done using revman (review manager 5.2). A priori, it was decided that only if the studies are sufficiently homogeneous, outcomes would be reported as pooled effect sizes. Studies reporting only continuous outcome measures, and also studies which were substantially heterogeneous and did not fit into a particular comparison group, were analysed separately and reported, without inclusion for the pooled effect estimate. To accommodate for any unexplained heterogeneity, random effects model was used for analysis. Statistical heterogeneity was calculated using Chi Square and also the *I* ^2^ statistic to describe the percentage variability in individual effect estimates that could be due to true differences between the studies rather than a sampling error. We considered *I* ^2^ < 40% as low, 30–60% as moderate, and >50% as substantial [6]. Further, study findings have also been shown in the form of “summary of findings” table, using the GRADE (Grading of Recommendations Assessment, Development, and Evaluation) approach. The utility of “an estimate of the magnitude of intervention effect” depends upon our confidence in that estimate. GRADE incorporates the aspects of study limitations, inconsistency of results, indirectness of the evidence, imprecision, and the reporting bias [7].

## 3. Results

### 3.1. Study Selection (PRISMA Flow Chart—Figure 1)

**Figure 1:**
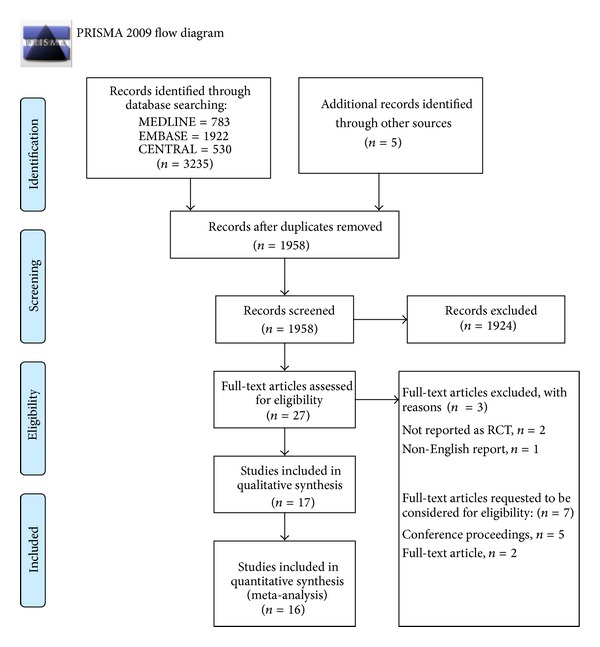
PRISMA flow diagram. From Moher D, Liberati A, Tetzlaff J, Altman DG, The PRISMA Group (2009). Preferred reporting items for systematic reviews and meta-analyses: The PRISMA Statement. PLoS Med 6(6): e1000097. doi:10.1371/journal.pmed1000097. For more information, visit http://www.prisma-statement.org.

The search results are highlighted as a flow diagram in Figure 1. Out of 3240 items, 1958 reports were obtained after removing duplicates. Finally, 27 full-text articles were considered for inclusion out of which 17 were included (Table 3) [8–24], and three were excluded [25–27]. Of the remaining seven studies [28–34], two were journal reports and five were conference proceedings. Despite multiple attempts, we could not obtain any full study report for the above seven studies. The study selection agreement between the authors was 0.73 using quadratic kappa weighting. For quantitative analysis (meta-analysis), only 16/17 studies were included; Hannallah and colleagues reported their results only as continuous outcomes [12].

### 3.2. Characteristics of Included Studies

Other important considerations are as follows. The study by Fisher and colleagues [9] included a three-arm design with caudal compared to inguinal nerve block. It involved the use of “epinephrine with LA” in only one group of CB. For the purpose of this review, both caudal groups were combined for comparisons, as suggested by [35]. The study by Tug and colleagues could not be considered appropriate under any comparator category and was hence reported separately [24]. Jahromi and colleagues compared caudal with two different groups [17]: infiltration and acetaminophen suppository. We included only the patients compared under infiltration with the caudal group. There was only one included study using US image guidance for INB [8].

### 3.3. Risk of Bias within Studies

**Figure 2:**
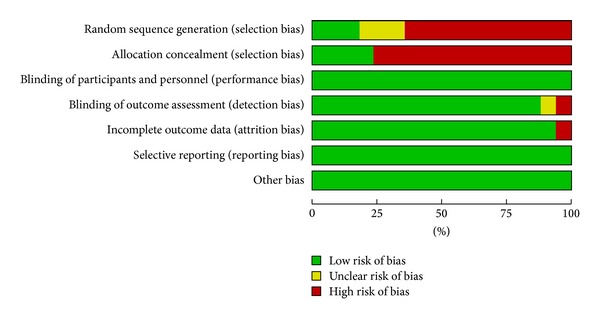
Risk of bias across studies assessed using the Cochrane risk of bias tool.

**Figure 3:**
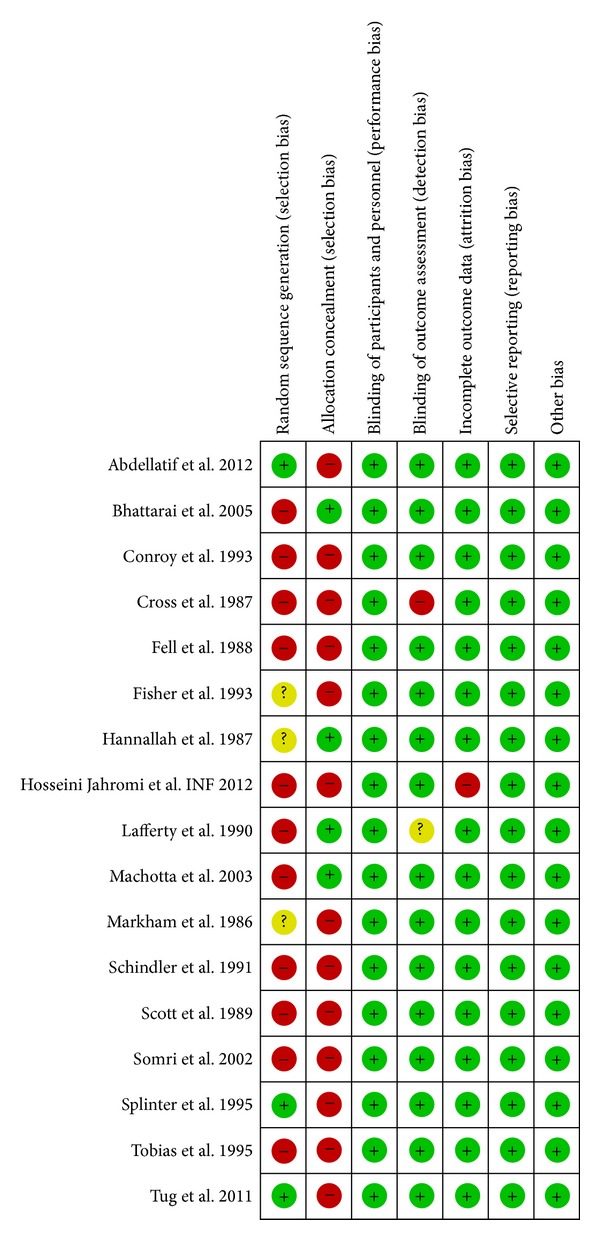
Risk of bias in individual studies using Cochrane risk of bias.

**Figure 4:**
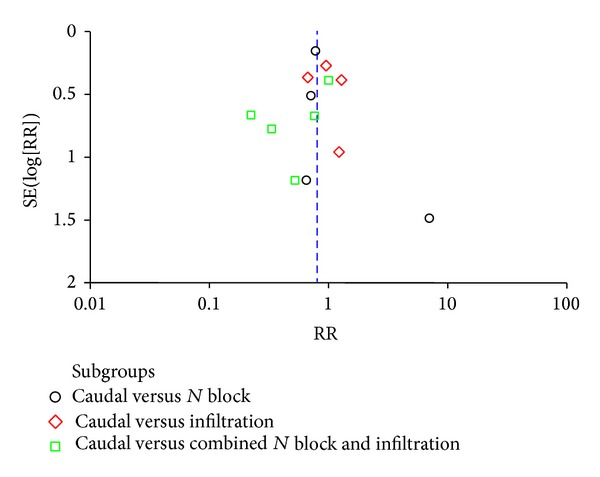
Funnel plot to identify the presence of publication bias.

The risk of bias across studies is represented in the bar graph obtained through revman (Figure 2). The risk of bias in individual studies, in specific domains, is shown in Figure 3. A majority of studies were observed as having a high risk of selection bias. Only seven studies reported the method used for sequence generation, and only five studies reported the method used for allocation concealment. Four studies excluded patients with failed interventions from the final analysis [8, 9, 17, 24]. For primary outcome analysis, we imputed the outcome of these excluded patients. Our rationale was that failed interventions will always necessitate rescue analgesic. Conroy and colleagues reported that they did not follow the randomisation sequence appropriately as generated for the first 30 patients [16]. Hence, we decided that a sensitivity analysis is to be carried out by excluding this study and observing the change in estimate of effect. The reporting of methodology and outcome assessment was not entirely clear in the study by Lafferty et al. [14]. We could not identify any major publication bias (funnel plot—Figure 4) and no study mentioned any specific funding support.

### 3.4. Outcome Analysis and Results 

#### 3.4.1. Use of Rescue Analgesia in Early Period: ≤4 hrs (Figure 5)

In total there were 14 studies with 851 patients. Tug and colleagues compared CB with single shot lumbar (L2) PVB [24]. We noted that the inclusion of this particular study resulted in heterogeneity and significant subgroup differences (test for subgroup differences: Chi = 7.66, df = 3 (*P* = 0.05), *I* ^2^ = 60.8%). Also, in practice it is not commonly performed for inguinal surgeries in children. After its exclusion, we had 13 studies with 789 children with the overall pooled estimate favouring caudal; RR: 0.81 [0.66, 0.99], *P* = 0.04, with no identifiable heterogeneity (*I* ^2^ = 0) or subgroup differences. The ARR (absolute risk reduction) was 1.38. Quality of evidence, according to the GRADE, is moderate (Table 2).

#### 3.4.2. Use of Rescue Analgesia in Late Period: 4–24 hrs (Figure 6)

In total there were 9 studies with 597 patients. Excluding one study [24], for reasons of heterogeneity, resulted in 8 studies with 532 children. Overall pooled estimate favours the benefit of analgesia from caudal; RR: 0.81 [0.69, 0.96], *P* = 0.01, reaching statistical significance. The ARR was 7.8. Quality of evidence, according to the GRADE-SOF, is moderate (Table 2).

#### 3.4.3. Side Effects

**Figure 7:**
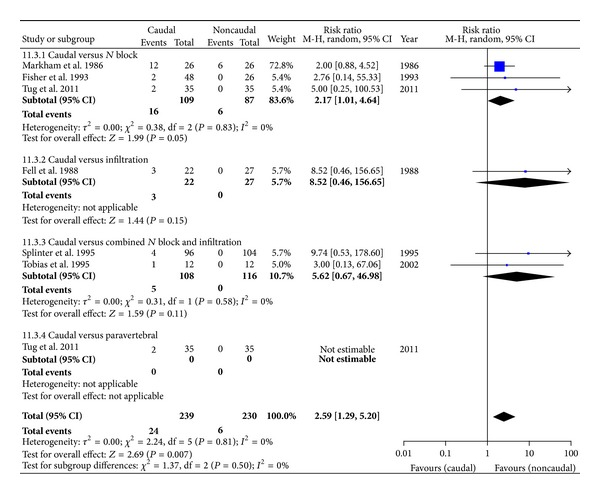
Forest plot for the incidence of motor block.

**Figure 8:**
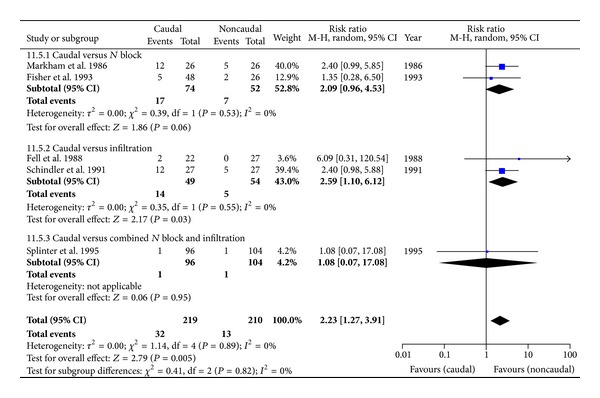
Forest plot for the incidence of urinary retention.

Motor blockade (Figure 7) was observed in 24/239 children in the CB group compared to 6/230 children in the comparator group: 6 studies with 469 children; RR = 2.59 [1.29, 5.20], *P* = 0.007. Urinary retention (Figure 8) was observed in 32/219 children in the CB group compared to 13/210 children in the comparator group: 5 studies with 459 children: RR = 2.23 [1.27, 3.91], *P* = 0.005. NV was observed to be similar in both caudal and noncaudal groups. Only Lafferty and others reported infection in one child belonging to the INF [14]; and only Fell and others reported delayed discharge in three and one, respectively, in CB and INF [15]. Although there were clearly more side effects with CB, the quality of evidence, according to GRADE, was very low, except for NV. Reasons for downgrading the evidence is provided within the SOF table (Table 2).

### 3.5. Description of Results within Individual Subgroups (Table 1)

#### 3.5.1. CB versus INB

We identified five studies, out of which four were included in the meta-analysis. Except Hannallah and colleagues (orchidopexy only) [12], the studies included patients from inguinal hernia and orchidopexy surgeries. All used bupivacaine in the concentration ranging from 0.2% to 0.5%. The volume injected ranged from 0.7 to 1 mL kg^−1^ (CB) and from 0.1 mL to 0.4 mL kg^−1^ (INB). Only Fisher and colleagues [9] used epinephrine mixed with bupivacaine in one arm of their caudal patients. We combined them together as belonging to CB. All except one performed both their interventions before surgery [9]. Hannallah and colleagues reported their pain scores only in “median range,” caudal (1.0, 6) and N block (1.0, 6), and did not report the use of rescue analgesic in the two groups separately [12].

#### 3.5.2. CB versus INF

We identified 6 studies in total, but only two studies [9, 12–15, 17] provided analgesia outcomes for both time periods. Both interventions were performed after surgery in two studies [13, 14, 17]; however the other 4 studies performed caudal preoperatively and infiltration postoperatively [14–16, 18]. Except for Lafferty and colleagues (only orchidopexy) [14], all included hernia surgeries only. All used bupivacaine in a concentration of 0.25% for CB and 0.25%–0.5% for INF. The volume ranged from 0.7 to 1.0 mL kg^−1^ (CB) and from 0.2 to 0.7 mL kg^−1^ (INF). Only Conroy and colleagues used epinephrine along with bupivacaine [16]. Variations of the infiltration techniques involved infiltration of the wound site through the skin and infiltration of fascia or aponeurosis before closure. No study used image guidance.

#### 3.5.3. CB versus Combined INB and INF

Five studies were identified. Tobias and colleagues also performed a laparoscopic inspection of the other side [19]. The studies contained a mix of hernia and orchidopexy surgeries. Except Bhattarai and colleagues [23], all placed their CB before surgery. However, the timing of INB and INF was variable. Epinephrine was used in three of the studies along with bupivacaine. Compared to other groups, bupivacaine concentration used was 0.25% in all studies except 0.2% for CB by Splinter and colleagues [20]. The volume ranged from 1 to 1.25 mL kg^−1^    (CB) and from 0.3 to 1 mL kg^−1^ (INB and INF).

#### 3.5.4. Caudal versus Others

Tug and colleagues used a single shot lumbar PVB to compare with CB for inguinal surgeries [24]. Out of 70 patients, six patients had a failed block (two in PVB and four in CB), and 12/35 patients in CB and 4/35 patients in PVB needed rescue analgesia during the early period with a RR: 3.0 [1.07, 8.04]. They also observed 2 cases of motor block in CB compared to 0 in PVB, out of 35 patients in each group.

*Additional Analysis (Sensitivity Analysis)*. Although we considered concentration of LA, timing of interventions as related to surgery, and the use of image guidance as potential sources of heterogeneity, we did not have sufficient number of studies to carry out further subgroup analysis.

(1) Conroy and colleagues had noted that the randomisation was not done appropriately for the first 30 patients [16]. Sensitivity analysis showed that the pooled effect size for the overall estimate and the subgroup (CB versus INF) estimate was not affected much for the early use of rescue analgesia.

(2) Ultrasound guided procedures: our search revealed only 2 studies [8, 29], out of which only a single study report was accessible. Abdellatif compared US-INB block with blind CB in children having inguinal hernia surgeries [8]. Average pain scores and use of rescue medications were not found to be significantly different. Use of rescue analgesia: early period: 5/25 (CB) and 7/25 (INB); late period: 9/25 (CB) and 8/23 (INB).

## 4. Discussion

### 4.1. Summary of Evidence

Our results show that CB is superior compared to the group of noncaudal regional analgesic interventions involving INF, INB, or their combination, demonstrated by the significantly reduced need for rescue analgesic during both early and late periods. However, the ARR (absolute risk reduction) was only 1.58 for the early period, compared to 7.94 for the late period, indicating that the benefits are perhaps more appreciable in the later period. Among the side effects, motor block and urinary retention were significantly more common with the caudal group with an ARR of 7.44 and 8.42, respectively. NV was found to be similar. Individually, among the subgroups, the need for rescue analgesia was less with CB compared to INB, and the combined INB with INF. However, the reduction did not achieve statistical significance.

For provision of postoperative pain relief in inguinal surgeries in children, regional procedures are preferred because of several advantages over parenteral analgesics [36]. Caudal analgesia has been widely used, and, because of the ease of administration, it is the most commonly used neuraxial block for children [37]. In children, most regional procedures are done under GA or heavy sedation [38]. The relative risks and benefits of CB as compared to less demanding techniques such as INB and infiltration are unclear. Complications could arise as a result of LA used or because of the nature of the regional technique [39]. The potential for harm is perhaps more with a neuraxial block. Our review shows that, despite the common practice of CB, there are limited studies. The exclusion of reports which used adjuvants did not seem to affect the study results. All of those excluded study reports, except a single study [40], had studied the effect of adjuvants when used in the caudal space without actually comparing it with other comparator regional techniques. The excluded study was a pilot study by Ivani and colleagues who studied the use of ropivacaine mixed with clonidine and compared between CB and INB, with children aged 1–7 years undergoing inguinal surgeries. The pain scores were similar, with 6/20 and 11/20 children needing rescue analgesia in INB and CB, respectively [40]. A systematic review also did not find convincing evidence for the use of nonopioid additives in elective outpatient surgery involving children [41].

In our review, most studies suffered from the risk of selection bias or did not specify the method of sequence generation and allocation concealment. This finding was similar, with studies published recently as well as in the past. Studies also suffered from smaller sample sizes. A majority of subjects (45%) came from the subgroup of CB against the combined group (355/789). Interestingly, the results in this subgroup favoured caudal for both early and late periods, while the individual subgroup comparisons of “CB against INB or INF” demonstrated similar effectiveness. It is difficult to reason or speculate on this observation. More studies with bigger sample sizes could potentially reveal the true differences. Although no direct comparison of “INF against INB” was done in our review, some studies have shown that the effectiveness of each could be similar. Both CB and INB have the potential to block the nerves of lower limb [42]. Our review observed that MB and UR are certainly more common with the CB than the INB, but their assessment suffered from lack of use of uniform, reliable, and validated criteria. The assessment of voiding difficulty requires the control of several confounders: hydration status, administration of agents during GA [9]. Higher incidence of MB and UR was seen particularly with two studies. Markham and colleagues observed 12/26 (CB) versus 6/26 (INB) children, found not walking at 6 hrs [10]. They also had a higher incidence of UR with 12/26 (CB) versus 5/26 (INB), having not voided at 6 hrs. It is possibly because of the higher concentration of the LA used, 0.5% as compared to others who used 0.25%. Schindler and colleagues reported 12/27 (CB) versus 6/27 (INF) children having not voided at the time of discharge (considered around 4 hrs in their study) [18]. CB against INB accounted for 228 patients among which the study by Fell and colleagues [15] accounted for a majority (82 patients).

Compared to INF, both CB and INB need more skill and both are operator dependent [5]. The risk of technical failure exists with both techniques. INB can also suffer from a success rate of only 70%–80% [43]. The use of US could potentially improve the precision of both CB and INB. It has been shown that the success rate of caudal injection [44, 45], as well as INB [4], could be better using US guidance. Despite this, we only found 2 studies comparing US-INB to CB [8, 29]. Although not directly applicable to our results, we explored for other studies on the possible use of US-INB in children. Apart from Willschke and colleagues [4], we only found 4 others. Two of them looked at the exact site of injection and plasma levels of ropivacaine, respectively [46, 47]. Another study looked at the addition of US guided INB with CB. [48]; pain scores were found to be significantly different; however, the amount of rescue analgesic used was not. Ghani and colleagues compared US-INB with US-TAP block and found that US-INB was superior [49]. Although it is acceptable to appreciate the superior technical efficiency of INB using US guidance, given the limited evidence, it cannot be extrapolated to infer a superior clinical effectiveness as compared to CB for inguinal surgeries in children. One must also keep in mind that the plasma levels of LA were found to be significantly higher with US guided blocks than landmark-based [47]; it has significant implications on doing a rescue block or any additional local infiltration.

### 4.2. Limitations

There were fewer studies, mostly with smaller sample sizes. Most studies were rated high for selection bias. There were no uniform, reliable, and validated outcome measures and the thresholds used for providing rescue analgesia were variable. It can also be argued that a network meta-analysis or multiple treatment comparison would have been a better approach. However, there are limitations to interpretation or inferences drawn from such an analysis as they could be prone to a higher degree of heterogeneity and invalid conclusions [50].

## 5. Conclusions

Caudal block provides superior analgesia requiring less rescue analgesic, with higher chances of motor block and urinary retention. There seems to be little advantage of combining both INB and INF as compared to CB; by requiring more volume, this may even cause harm by potentially increasing the chances of LA toxicity. As shown in the attached SOF table, the evidence level for the analgesic requirement is moderate and future studies looking to evaluate this comparison will have an important impact on the confidence of this estimate. More comparative studies are required to demonstrate that better technical efficiency, with the use of US-INB, translates into superior clinical effectiveness, as compared to CB alone. Apart from larger sample sizes, studies should use well defined criteria for measurement of these outcomes.

## Figures and Tables

**Figure 5:**
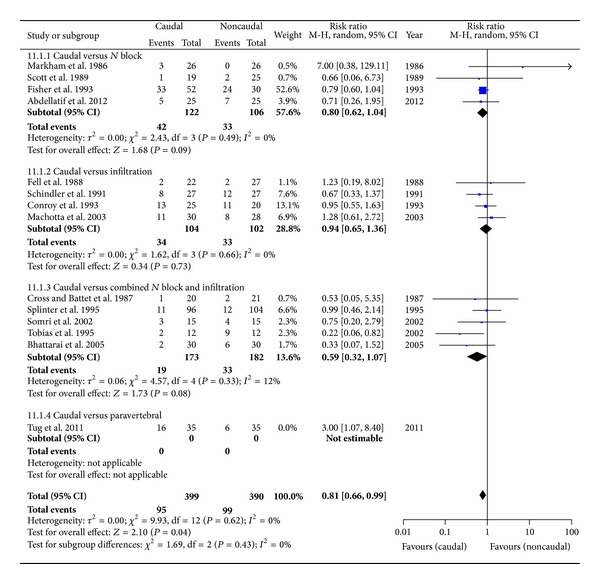
Forest plot for the use of rescue analgesia in the early period.

**Figure 6:**
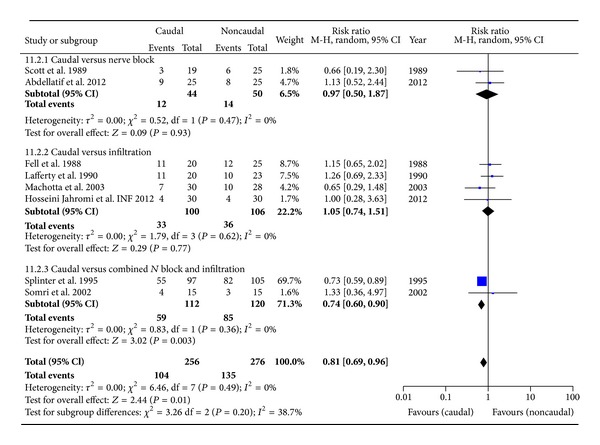
Forest plot for the use of rescue analgesia in the late period.

**Table 1:** Summary of pooled outcomes in subgroups.

Group	Number of studies and children	Outcome	Remarks
Outcome: early rescue analgesia (up to 4 hrs)			
CB versus INB	4 studies: CB: 122 INB: 106	RR: 0.80 [0.62, 1.04]	*I* ^2^ = 0 No significant difference
CB versus INF	4 studies: CB: 104 INF: 102	RR: 0.94 [0.65, 1.36]	*I* ^2^ = 0 No significant difference
CB versus combined	5 studies: CB: 173 Combined: 182	RR: 0.59 [0.32, 1.07]	*I* ^2^ = 0 No significant difference

Outcome: late rescue analgesia (4–24 hrs)			
CB versus INB	2 studies: CB = 44, INB = 50	RR: 0.97 [0.50, 1.87]	*I* ^2^ = 0 No significant difference
CB versus INF	4 studies CB = 100, INF = 106	RR: 1.05 [0.74, 1.51]	*I* ^2^ = 0 No significant difference
CB versus combined	2 studies CB = 112 and combined = 120	RR: 0.74 [0.60, 0.90], *P* = 0.003	*I* ^2^ = 0 ARR = 17.4% Significantly favouring caudal

Outcome: motor block			
CB versus INB	3 studies: CB = 109, INB = 87	RR: 2.17 [1.01, 4.64] *P* = 0.05	*I* ^2^ = 0 Motor block more common with CB ARR = 7.2%
CB versus INF	1 study: CB = 22, INF = 27	Event rate: 3/22 (CB) 0/27 (INF)	Only 1 study; motor block not observed with INF
CB versus combined	2 studies: CB = 108 and combined = 116	RR: 5.62 [0.67, 46.98]	*I* ^2^ = 0 All 5 patients noted to have a motor block belonged to CB

Outcome: vausea-vomiting			
CB versus INB	2 studies: CB = 50, INB = 49	RR: 0.57 [0.18, 1.80]	*I* ^2^ = 0 No significant difference
CB versus INF	2 studies: CB = 49, INF = 54	RR: 0.77 [0.36, 1.64]	*I* ^2^ = 0 No significant difference
CB versus combined	3 studies: CB = 146 and combined = 154	RR: 1.13 [0.86, 1.50]	*I* ^2^ = 0 No significant difference

Outcome: urinary retention			
CB versus INB	2 studies: CB = 74, NB = 52	RR: 2.09 [0.96, 4.53] *P* = 0.06	I^2^ = 0, favouring INB, but not significant
CB versus INF	2 studies: CB = 49 INF = 54	RR: 2.59 [1.10, 6.12], *P* = 0.03	*I* ^2^ = 0: ARR = 19.3% Significantly favouring INF
CB versus combined	1 study: CB = 96, combined = 104	Event rate: 1/96 (CB) 1/104 (combined)	Only 1 study

**Table 2:** Summary of findings using GRADE **(**Grading of Recommendations Assessment, Development, and Evaluation) approach.

Caudal compared to noncaudal regional analgesia for inguinal surgeries in children						
*Patient or population*: patients with inguinal surgeries in children *Settings*: randomised control studies reported in English language *Intervention*: caudal *Comparison*: voncaudal regional analgesia						

*Outcomes *	*Illustrative comparative risks* ∗ * (95% CI) *		*Relative effect* *(95% CI) *	*No. of Participants* *(studies) *	*Quality of the evidence* *(GRADE) *	*Comments *
	Assumed risk	Corresponding risk				
	*Noncaudal eegional analgesia *	*Caudal *				

*Early rescue analgesia (<4 hrs)* Number of children Needing rescue analgesic Medication	*Study population *		*RR 0.81* (0.66 to 0.99)	789 (13 studies)	*⊕⊕* *⊕⊝* *mo* *de* *ra* *te* ^1,2,3^	
	*254 per 1000 *	*206 per 1000* (168 to 251)				
	*Moderate *				
	*233 per 1000 *	*189 per 1000* (154 to 231)			

*Late rescue analgesia (4 hrs–24 hrs)* Number of children Needing rescue analgesic Medication	*Study population *		*RR 0.81* (0.69 to 0.96)	532 (8 studies)	*⊕⊕* *⊕⊝* *mo* *de* *ra* *te* ^1,2,3^	
	*489 per 1000 *	*396 per 1000* (338 to 470)				
	*Moderate *				
	*339 per 1000 *	*275 per 1000* (234 to 325)			

*Adverse effect-motor blockade *	*Study population *		*RR 2.68* (1.36 to 5.28)	539 (6 studies)	*⊕⊝* *⊝⊝* *ve* *ry* *low* ^1,2,3,4,5,6^	
	*23 per 1000 *	*61 per 1000* (31 to 120)				
	*Moderate *				
	*0 per 1000 *	*0 per 1000* (0 to 0)			

*Adverse effect-nausea-vomiting *	*Study population *		*RR 1.05* (0.81 to 1.35)	502 (7 studies)	*⊕⊕* *⊕⊝* *mo* *de* *ra* *te* ^1,2,3^	
	*261 per 1000 *	*274 per 1000* (211 to 352)				
	*Moderate *				
	*222 per 1000 *	*233 per 1000* (180 to 300)				

*Adverse effects-urinary retention *	*Study population *		*RR 2.23* (1.27 to 3.91)	429 (5 studies)	*⊕⊝* *⊝⊝* *ve* *ry* *low* ^1,2,3,6,7,8^	
	*62 per 1000 *	*138 per 1000* (79 to 242)				
	*Moderate *				
	*77 per 1000 *	*172 per 1000* (98 to 301)			

*Adverse effects-delayed discharge *	*Study population *		*RR 3.68* (0.41 to 32.97)	49 (1 study)	*⊕⊝* *⊝⊝* *ve* *ry* *low* ^5,6,9,10^	
	*37 per 1000 *	*136 per 1000* (15 to 1000)				
	*Moderate *				
	*37 per 1000 *	*136 per 1000* (15 to 1000)			

*Adverse effects-infection-caudal versus infiltration *	*Study population *		*RR 0.38* (0.02 to 8.86)	43 (1 study)	*⊕⊝* *⊝⊝* *ve* *ry* *low* ^3,5,6,9,10^	
	*43 per 1000 *	*17 per 1000* (1 to 385)				
	*Moderate *				
	*44 per 1000 *	*17 per 1000* (1 to 390)			

*The basis for the *assumed risk* (e.g., the median control group risk across studies) is provided in footnotes. The *corresponding risk* (and its 95% confidence interval) is based on the assumed risk in the comparison group and the *relative effect* of the intervention (and its 95% CI).

*CI*: confidence interval; *RR*: risk ratio.

GRADE Working Group grades of evidence:

*high quality*: further research is very unlikely to change our confidence in the estimate of effect;

*moderate quality*: further research is likely to have an important impact on our confidence in the estimate of effect and may change the estimate;

*low quality*: further research is very likely to have an important impact on our confidence in the estimate of effect and is likely to change the estimate;

*very low quality*: we are very uncertain about the estimate.

^1^There was no appropriate concealment in the majority of the studies.

^2^Based on available studies, funnel plot looks symmetrical.

^3^None of the studies were industry funded.

^4^No uniform criteria were considered for assessment of motor blockade.

^5^Wide confidence interval.

^6^Sample size too low to detect a true difference.

^7^No uniform criteria used for assessment of urinary retention.

^8^Several confounders were not controlled appropriately.

^9^No appropriate concealment or random sequence generation.

^10^Only a single study.

**Table 3:** Characteristics of included studies.

Author, year, and methods	Participants	Interventions	Outcomes	Notes
Caudal versus inguinal nerve block				

Abdellatif 2012 [8] RCT, 2 groups, parallel design	Children with unilateral groin surgery Age: 1–6 yrs	US guided INB against blind CB; both done preoperatively under GA. No use of adrenaline.	CHEOPS scale and also the number of children needing rescue analgesic provided.	1 patient in CB and 2 in INB were excluded due to failure.

Fisher et al., 1993 [9] RCT, 3 groups, parallel design	Children having herniorrhaphy or orchidopexy Age: 0.5–10 yrs	2 groups of CB (with or without the use of epinephrine) against INB; both done after the procedure.	Primary outcome: postoperative voiding with analgesia outcomes as secondary.Single time point reporting of rescue analgesia.	For the purpose of the review the caudal groups were combined as 1 group. 4 patients in each group were excluded because of failure of interventions.

Markham et al., 1986 [10] RCT, 2-arm parallel trial	Children having herniorrhaphy or orchiopexy Age: 1–12 years	CB against INB; both done preoperatively, without image guidance under GA. No use of adrenaline.	The outcome was intraoperative and postoperative analgesia.	

Scott et al., 1989 [11] RCT, 2-arm parallel trial	Children having herniorrhaphy or orchiopexy Age: 3–8 years	CB against INB; both done preoperatively, without image guidance under GA. No use of adrenaline.	Primary outcome: effectiveness of postoperative analgesia.	

Hanallah et al., 1987 [12] RCT, 3-arm parallel trial	Orchidopexy Age: 18 months–12 years	CB against INB, with the 3rd group acting as a control. All interventions done after surgery, without image guidance No use of adrenaline.	Primary outcome: postoperative analgesia as median and range without specifying the time point.	Not included in the quantitative analysis. The authors also combined both treatment groups compared to the control group to report the use of rescue analgesia.

Caudal versus infiltration				

Machotta et al., 2003 [13] RCT, 2-arm parallel trial	Children having unilateral Hernia Age: 0–5 yrs	CB against wound infiltration; both done after the surgery. No Image guidance or use of epinephrine.	Postoperative analgesia. Hannalah scale as well as children needing rescue analgesic.	Adverse events are not specifically (individually) reported.

Lafferty et al., 1990 [14] RCT, 2-arm parallel design	Children having orchiopexy Age: 2–15 years	CB done preoperatively versus wound infiltration done before full surgical closure. No image guidance or use of epinephrine.	Postoperative analgesia by a 10 cm linear analogue scale and use of rescue analgesia.	Poor reporting of methods and outcome assessment

Fell et al., 1988 [15] RCT, 2-arm parallel design	Children having inguinal herniotomy Mean age CB: 4.5 ± 2.9 yrs INF: 3.7 ± 2.5 yrs	Caudal done preoperatively versus wound infiltration after surgery. No image guidance or use of epinephrine	Analgesia rated on a 3-point scale. Proportions of patients who were pain free provided.	Calculation of the number of children needing rescue analgesic was done indirectly. 1 patient was excluded as the data was incomplete.

Conroy et al., 1993 [16] RCT, 3-arm parallel trial, with a control group.	Children having a bilateral inguinal hernia Age: 2 months–10 years	CB done preoperatively versus INF after surgery. No image guidance. Epinephrine used in both groups.	Postoperative analgesia. Specific time point used to calculate the number of rescues analgesic not clearly mentioned.	Children in the control group were not included in this review. Confusion in the randomization code, in the first 30 pts, led to more children having caudal blocks.

Jahromi et al., 2012 [17] RCT, 3-arm parallel design	Unilateral inguinal hernia Age: 0.3–7 years	Caudal versus INF, both done after the surgery. No image guidance or epinephrine was used. 3rd group of acetaminophen was not included	Analgesia in FLACC scale and also reported as the number needing rescue analgesic.	3 children in the caudal group were excluded because of failed caudal.

Schindler et al., 1991 [18] RCT, 2-arm parallel design	Unilateral inguinal hernia Age: 2 months–12 years	CB done preoperatively versus INF done before full surgical closure. No image guidance or epinephrine used.	Analgesia in CHEOPS scale and also reported as the number needing rescue analgesia.	

Caudal versus combined wound infiltration and inguinal N block				

Tobias et al., 1995 [19] RCT, 2-arm parallel design	Children having inguinal hernia with additional laparoscopic inspection of contralateral peritoneum Mean age CB: 1.2 ± 0.2 yrs Comparator: 1.3 ± 0.4 yrs	CB placed presurgically versus INB and INF. No image guidance. Epinephrine used in both arms.	Analgesia using Hannalah scale and also reported as the number needing rescue analgesia.	Laparoscopic inspection involved.

Splinter et al., 1995 [20] RCT, 2-arm parallel design	Children having inguinal hernia repair Age: 1–13 years	CB placed presurgically versus INB and INF placed after surgery. No image guidance. Epinephrine used in both arms.	Analgesia using mCHEOPS scale and also reported as the number needing rescue analgesic.	

Cross and battett 1987 [21] RCT, 2-arm parallel design	Children having herniotomy or/and orchidopexy; unilateral or bilateral included. Age: 1–13 years	CB versus INB and INF, all placed before surgery. No image guidance. Epinephrine used only in the comparator group.	Analgesia using linear analogue scale and also reported as the number needing rescue analgesic.	The dose of local anesthetic was different depending on unilateral and bilateral surgeries.

Somri et al., 2002 [22] RCT, 2-arm parallel design	Children having orchidopexy Age: 1–8 years	CB versus combined INB and INF. No image guidance or use of epinephrine.	Primary outcome-effect of catecholamine level. Analgesia as a secondary outcome, reported as the number needing rescue analgesic.	The report is titled as a comparison of CB versus INB; however the methods mention that they supplemented the INB with INF.

Bhattarai et al., 2005 [23] RCT, 2-arm parallel design	Children having herniotomy Age: 1–14 years	CB versus combined INB and INF; all interventions done after surgery. No image guidance used.	Analgesia reported as mean duration and also as the number needing rescue analgesic.	

Caudal versus others				

Tug et al., 2011 [24] RCT, 2-arm parallel design	Inguinal hernia Age: 3–7 years	CB versus PVB; both placed presurgically.	Rescue analgesia at 2 and 4 hrs and also in mean (±SD scores).	2 (PVB) and 4 (CB) were excluded due to technical failures.

(RCT: randomised control trial, CB: caudal block, INB: inguinal nerve block, and INF: infiltration).

## Appendix

### A. Search Strategy

#### A.1. MEDLINE: Up to Feb 20th 2013

1. exp hernioplasty/or exp inguinal hernia/or inguinal herni*.mp. or exp herniorrhaphy/
2. exp herniotomy/or herniotom*.mp.
3. herniorrhaph*.mp. [mp = title, abstract, subject headings, heading word, drug trade name, original title, device manufacturer, drug manufacturer, device trade name, keyword]
4. hernioplast*.mp. [mp=title, abstract, subject headings, heading word, drug trade name, original title, device manufacturer, drug manufacturer, device trade name, keyword]
5. hernia repai*.mp. [mp = title, abstract, subject headings, heading word, drug trade name, original title, device manufacturer, drug manufacturer, device trade name, keyword]
6. 1 or 2 or 3 or 4 or 5
7. exp caudal anesthesia/or exp epidural anesthesia/or caudal an*.mp.
8. epidural an*.mp.
9. caudal.mp.
10. epidural.mp.
11. exp ropivacaine/or exp bupivacaine/or exp local anesthetic agent/or bupivacaine.mp.
12. local anesthe*.mp. [mp=title, abstract, subject headings, heading word, drug trade name, original title, device manufacturer, drug manufacturer, device trade name, keyword]
13. 7 or 8 or 9 or 10 or 11 or 12
14. 6 and 13
15. orchidopexy.mp. or exp orchidopexy/
16. 6 or 15
17. 13 and 16.

#### A.2. EMBASE: Up to Feb 20th 2013

1. exp Herniorrhaphy/or exp Hernia, Inguinal/or inguinal herni*.mp.
2. herniorraph*.mp. [mp = title, abstract, original title, name of substance word, subject heading word, keyword heading word, protocol supplementary concept, rare disease supplementary concept, unique identifier]
3. Herniotom*.mp.
4. Hernioplast*.mp. [mp = title, abstract, original title, name of substance word, subject heading word, keyword heading word, protocol supplementary concept, rare disease supplementary concept, unique identifier]
5. hernia repai*.mp. [mp = title, abstract, original title, name of substance word, subject heading word, keyword heading word, protocol supplementary concept, rare disease supplementary concept, unique identifier]
6. 1 or 2 or 3 or 4 or 5
7. exp Anesthesia, Caudal/or caudal.mp.
8. caudal analges*.mp. [mp=title, abstract, original title, name of substance word, subject heading word, keyword heading word, protocol supplementary concept, rare disease supplementary concept, unique identifier]
9. caudal anesthesi*.mp. [mp=title, abstract, original title, name of substance word, subject heading word, keyword heading word, protocol supplementary concept, rare disease supplementary concept, unique identifier]
10. caudal bloc*.mp.
11. epidural.mp. or exp Anesthesia, Epidural/or exp Analgesia, Epidural/or exp Injections, Epidural/
12. local anestheti*.mp. or exp Anesthetics, Local/
13. bupivacaine.mp. or exp Bupivacaine/
14. ropivacaine.mp.
15. 7 or 8 or 9 or 10 or 11 or 12 or 13 or 14
16. 6 and 15
17. orchidopexy.mp. or exp Orchiopexy/
18. 1 or 2 or 3 or 4 or 5 or 17.
